# Multiple primary malignant neoplasm: Case report and comprehensive literature review

**DOI:** 10.3389/fonc.2022.1090634

**Published:** 2023-01-04

**Authors:** Xue-Yan Ma, Kun Tian, Peng-Fei Sun

**Affiliations:** ^1^ Department of Oncology, Second Clinical Medical College of Lanzhou University, Lanzhou, China; ^2^ Department of Radiotherapy, Second Hospital of Lanzhou University, Lanzhou, China; ^3^ Department of Medical Oncology, Second Hospital of Lanzhou University, Lanzhou, China

**Keywords:** multiple primary tumors, NGS, KRAS germline mutations, systemic therapy, literature review

## Abstract

Multiple primary tumors, especially quadruple primary tumors, are extremely rare clinically, and there is no standard protocol for clinical management. We described a case in which a bone tumor, a malignant bladder tumor, a malignant melanoma, and an intrahepatic cholangiocarcinoma were all original malignancies. The patient is a 79-year-old woman who underwent surgery for a left middle finger bone tumor 45 years ago, as well as surgery for bladder malignancy and postoperative adjuvant chemotherapy 15 years ago, and the precise pathological results and treatment are unclear. One year ago, she underwent amputation of the toe due to a black mass of the right toe and was diagnosed pathologically as a freckled malignant melanoma of the extremity. Prior to postoperative adjuvant systemic medication, PET/CT revealed malignancy in the lateral segment of the left lobe of the liver, and multiple lymphadenopathies in the left parotid gland, hilar hepatic, and retroperitoneal region. Intrahepatic cholangiocarcinoma was found in the liver puncture biopsy’s pathology report. The serum sample’s next-generation sequencing (NGS) revealed a missense mutation, designated P.G12V, in exon 2 of the KRAS gene. Based on patients with malignant melanoma and intrahepatic cholangiocarcinoma, she received 6 cycles of GP (gemcitabine/cisplatin) combined with Camrelizumab systemic therapy, and followed by 3 cycles of Camrelizumab maintenance therapy, the efficacy was evaluated as stable disease (SD) during treatment. When the 4th cycle of Camrelizumab was suggested for maintenance therapy, the efficacy evaluation revealed that the tumor had greatly advanced. The patient refused to continue anti-tumor therapy and passed away from septic shock and multiple organ failure 3 months later. The patient had satisfactory efficacy and lived for a year after being diagnosed with two primary cancers. Despite the rarity of quadruple primary tumors and the lack of a conventional clinical management strategy, we postulate that germline mutations in the KRAS gene may be closely associated with the formation and development of multiple primary tumors. NGS testing is necessary for clinical management, and systemic treatment based on concurrent multiple main tumors is the key to improving prognosis.

## Background

Hurt et al (1)first reported multiple primary malignant neoplasms (MPMNs) in 1921, which are defined as two or more primary malignant tumors occurring simultaneously or sequentially in a single or multiple organs of the same host, and each tumor does not represent tumor expansion, recurrence, or metastasis (2). With the advancement of tumor diagnosis and treatment technology in recent years, the clinical reports of MPMNs seem to be increasing. However, four primary tumors have yet to be recorded, owing to the lack of a uniform approach for the whole-course management of MPMNs. We described a case of intrahepatic cholangiocarcinoma, malignant melanoma, bone tumor, and bladder cancer as primary malignancies. Based on the coexistence of malignant melanoma and intrahepatic cholangiocarcinoma, the patient received systemic treatment using chemotherapy combined with immune checkpoint inhibitors. To investigate the whole-course management strategy of MPMNs with the goal of providing a reference for standardized diagnosis and treatment in MPMN patients.

## Case presentation

### Main complaints

The 79-year-old female patient was admitted to the hospital due to a black mass on the bigger toe of the right foot for more than 4 years, the radical excision of the skin lesion after completing a relevant investigation was done and diagnosed postoperatively pathologically as a freckled malignant melanoma. Amputation of the toe was performed after 10 days. she was admitted to the hospital again one month after surgery for adjuvant therapy. There have been no special symptoms such as loss of appetite, fatigue, shortness of breath, palpitations, and weight loss since this onset.

### Medical history

The patient has had hypertension for over 40 years with no history of diabetes, coronary heart disease, or tuberculosis; the most severe hypertension is 160/90mmHg, which is well controlled by oral antihypertensive medications. Underwent resection of bone tumor in left-hand middle finger 45 years ago, cholecystectomy for gallbladder stones 39 years ago, and resection of bladder malignant tumor followed by adjuvant chemotherapy15 years ago, the exact pathological diagnosis and systemic treatment are unknown at that time. Since then, the patient has lived in a good state without clinical manifestations of tumor recurrence and metastasis. Denying a family history of malignancy.

### Physical examination

There were no yellow stain, rash, or bleeding spots on the skin and mucosa of the whole body, and superficial lymph nodes were not touched. Surgical scars were seen on the right abdomen and the fingers of the left hand. The first metatarsophalangeal of the right foot was missing with a well-healed surgical incision.

### Laboratory tests

Serum tumor markers were detected as follows: CEA 184.00 ng/ml, CA125 778.00U/ml, CA199>1000.00U/ml, CA72-4 22.10U/ml, NSE 16.10ng/ml and CY211 11.0ng/ml. No significant abnormalities were found in other laboratory tests.

### Radiographic examination

**Figure 1:**
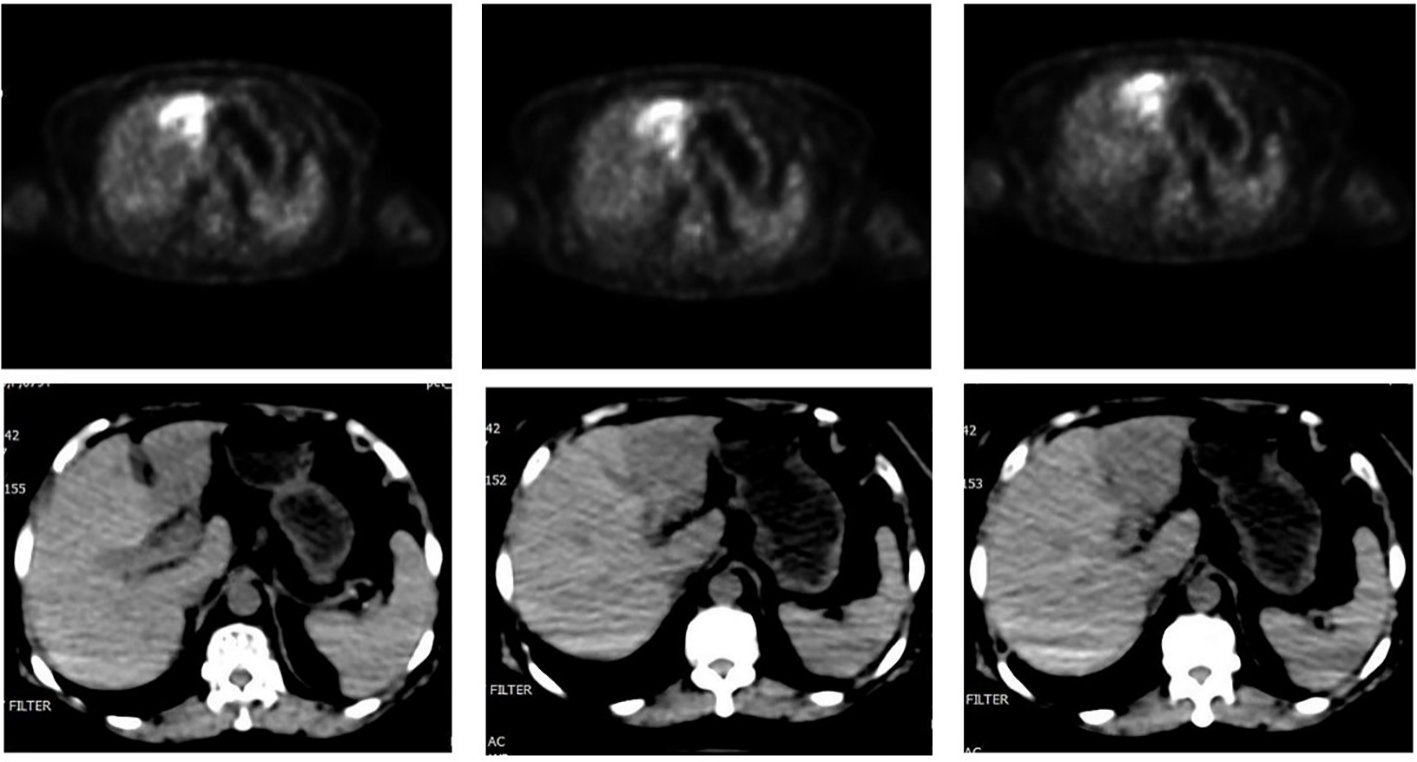
PET-CT revealed an irregular hypodense mass in the lateral segment of left lobe of liver with enhanced FDG drug absorption.

Prior to postoperative adjuvant therapy, a PET/CT (**Figure 1**) scan revealed a mild hypodensity mass with a maximum SUV value of 12.0 in the lateral segment of the left hepatic lobe, which was diagnosed as a malignant tumor. In addition, enlarged lymph nodes were visible in the left parotid gland, hilar hepatic and retroperitoneal regions, and FDG metabolism is elevated, which was considered as metastasis. CT scan showed a wedge-shaped and slightly hypodense mass in the lateral segment of the left hepatic lobe. MRI revealed an irregular soft tissue mass in the lateral segment of the left hepatic lobe with a slightly low signal intensity on T1WI and a heterogeneous slightly high signal intensity on T2WI, as well as dilated adjacent bile ducts.

### Pathological examination

**Figure 2:**
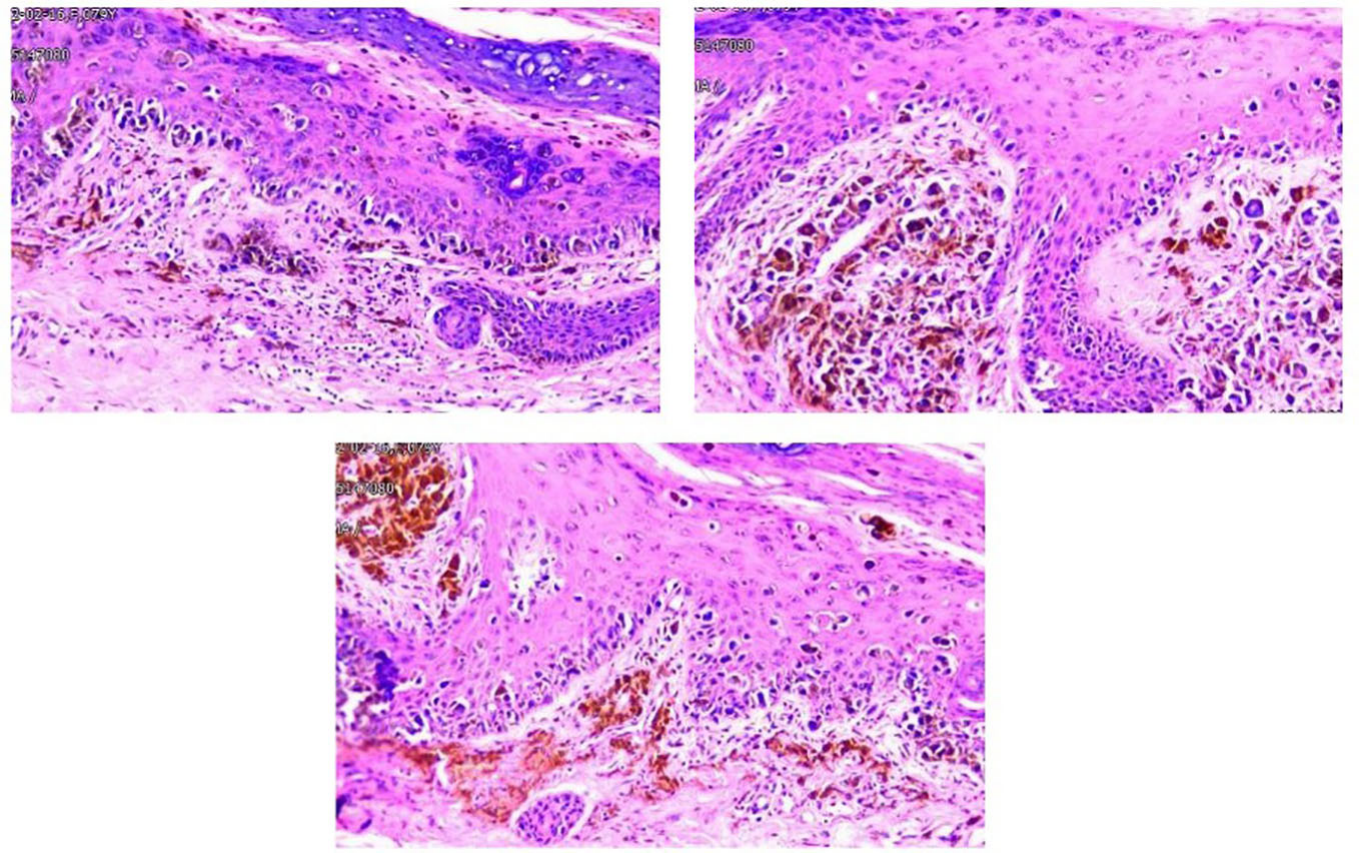
Tumor cells were found in the superficial dermis, the whole epidermis, and the inner corneal layer showed as nested sheets or scattered distribution. The ovoid and polygonal tumor cells had a distinct nuclear membrane, and an eosinophilic nucleolus, as well as abundant cytoplasm and melanin granules and an increased nucleoplasm ratio. Phagocytic melanocytes are found in the superficial dermis.

**Figure 3:**
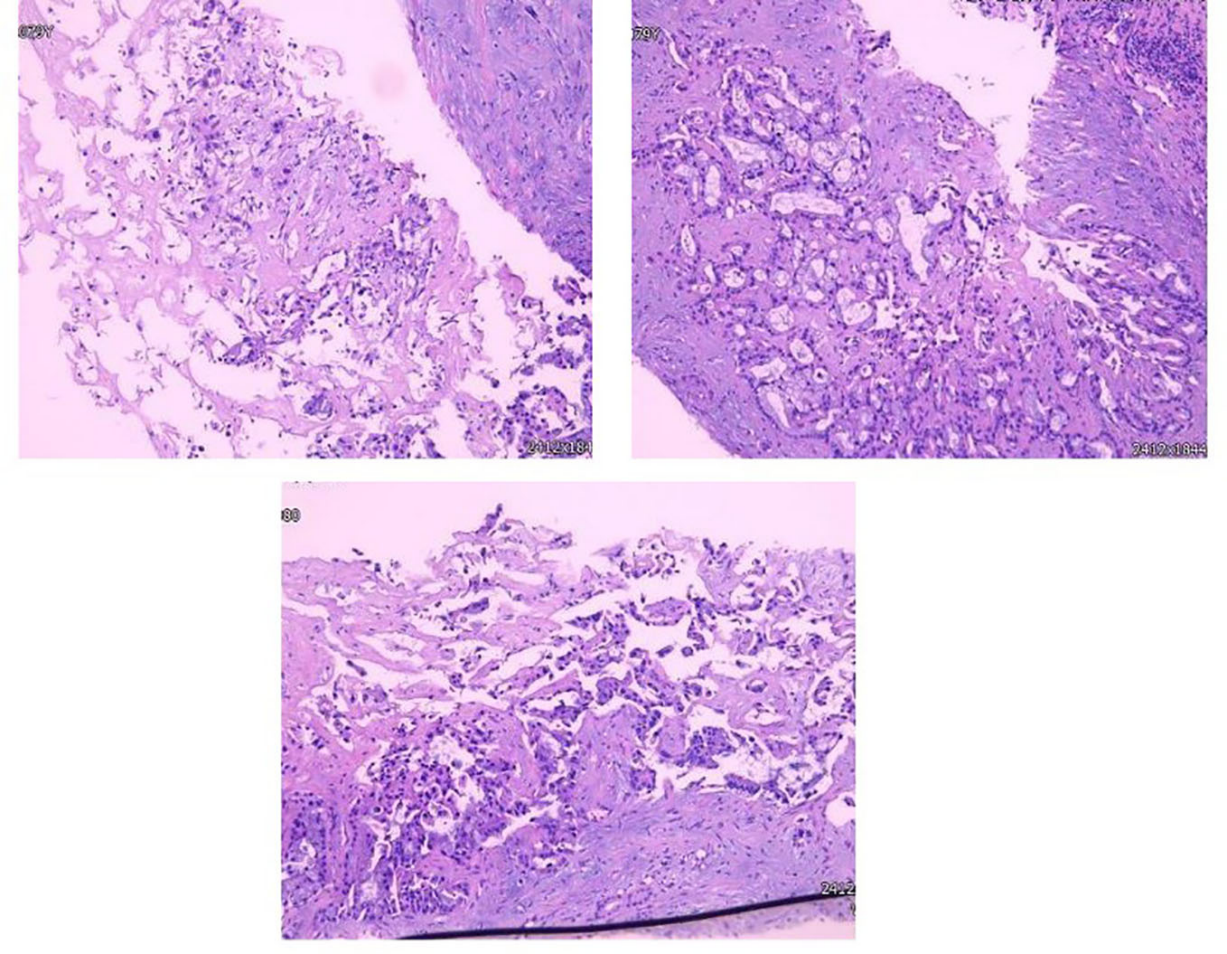
Glandular formations of varied size, shape, and irregular arrangement that have invaded the submucosa.

The resected mass of the right toe was submitted to undergo histology and immunochemistry. Histologically, the freckle-like tumor tissue invaded the superficial dermis with a 0.6 cm of maximum diameter and 0.2 mm Breslow thickness, which was identified as grade Clark IV without residual tumor cells in transverse and longitudinal margins. Pathological diagnosis of acral freckle-like malignant melanoma. Immunoperoxidase stains were performed on a Ventana NEXES Automated Immunohistochemistry System (Fuzhou Maixin Biotech Co., Ltd., Fuzhou, China) Vimentint, HMB45 and Melan A were positive in the tumor cells, while CKp, S-100, SOX-10, and CD68 were negative. Ki-67 was reactive in approximately 40% of the tumor cells (**Figure 2**). Pathological diagnosis is a freckled malignant melanoma of the extremities. Immunohistochemical features of liver aspiration biopsy tissue are as follows (**Figure 3**): CK19, CK7, CKp, and MUC5AC were positive in tumor cells, while Hepatocyte, CDX-2, TTF-1, HMB45, Melan-A, S100, and SOX10 were negative. The pathological diagnosis of a liver tumor is intrahepatic cholangiocarcinoma. Serum NGS testing suggested a potentially clinically significant missense mutation in exon 2 of the KRAS gene, namely P.G12V.

### Diagnosis

Based on medical history and double Primary Cancer confirmed this time, four primary tumors were comprehensively diagnosed, including bone tumors, bladder malignancies, malignant melanoma, and hepatic cholangiocarcinoma.

### Treatment

**Figure 4:**
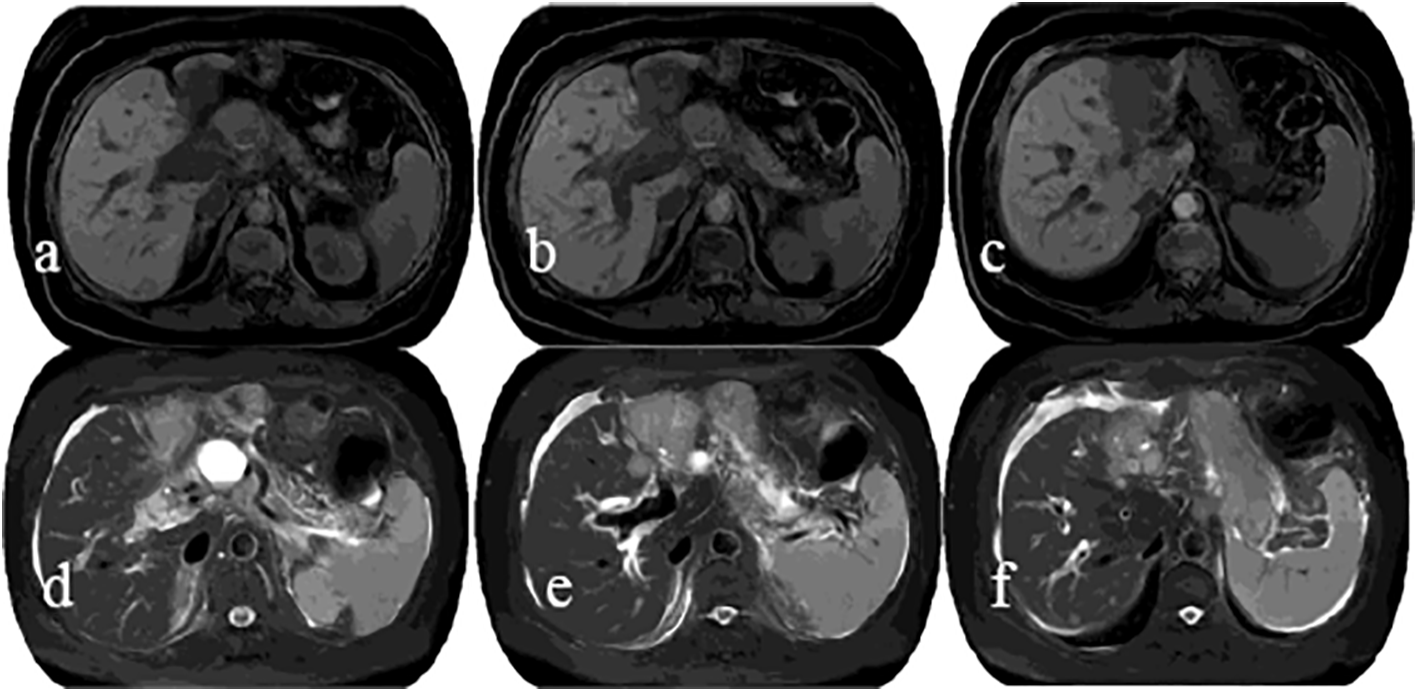
MRI showed an irregular soft-tissue mass with low signal intensity on T1WI **(A–C)** and slightly high signal intensity on T2WI **(D–F)** in the lateral segment of left hepatic lobe, as well as dilated adjacent bile ducts, and no clear indication of left portal vein.

Based on NGS (P.G12V) and coexisting Acral melanoma(AM) and intrahepatic cholangiocarcinoma as well as systemic lymph node metastasis, there is currently no sensitive Class A targeted drugs. 6 cycles of GP (gemcitabine hydrochloride 1.5g, d1,8/cisplatin 40 mg, d1, 8) combined with anti-PD-1 inhibitors (Camrelizumab 200 mg, d1) system therapy From April 2021 to August 2021, Followed by 3 cycles of Camrelizumab (200 mg, q3w) maintenance therapy during September to October 2021, and efficacy evaluation (RECIST version 1.1) as SD throughout treatment. she was re-admission because of mild yellowing sclera in December 2021, laboratory tests showed abnormal liver function indicators such as DBIL 40.1 umol/L, IBIL 48.1 umol/L, ALT 205 U/L, and AST 181 U/L. MRCP indicated (**Figure 4**) multiple liver metastases and biliary obstruction, and tumor progression was considered, so the 4th cycle of immune maintenance therapy was suspended. To improve liver function, endoscopic retrograde cholangiography (ERCP) and biliary stenting were also carried out. The tumor continued to progress 1 month after ERCP surgery, and the patient and family refused to continue anti-tumor therapy. Infectious shock and multiple organ failure caused the patient’s death in March 2022. The patient had a satisfactory curative effect and lived for up to a year after being diagnosed with double primary cancers.

## Discussion

According to Warren and Gates (3), multiple primary malignant neoplasms (MPMNs) are classified as contemporaneous MPMNs if there is less than a 6-month gap between two incidences and heterochronous MPMNs if there is a 6-month or longer gap. In the present patient, the intrahepatic cholangiocarcinoma was discovered incidentally during postoperative PET-CT evaluation of Acral melanoma, and the interval between the onset of the two primary malignancies could not be determined. Although a definitive diagnosis of MPMN could be made, simultaneous and heterochronic MPMNs could not be distinguished. The incidence of MPMNs has increased with the advancement of diagnostic techniques for malignant tumors and the prolongation of patient survival time, but cases with four primary tumors are still extremely uncommon. According to the PubMed database, the incidence of four primary tumors, including autopsy cases, was 0.007% (4), and only a very limited number of cases have been subjected to tumor NGS testing. Meanwhile, four primary tumors involved in bone tumors, malignant bladder tumors, intrahepatic cholangiocarcinoma, and Acral melanoma have not been reported.

RAS is the most frequently mutated oncogene in human malignancies and a key kinase in the RAS-RAF-MAPK signaling pathway, as well as taking part in other tumor-related signaling pathways such as PI3K/AKT, PAC, and PAL signaling pathways (5).Mutations in the AKT family have been found in up to 43-60% of melanoma cases (6). PTEN, which inhibits the PI3K/AKT/mTOR growth-promoting signaling cascade, is found in 38% of primary melanoma patients and 58% of metastatic disease patients (7). PTEN and BRAF pathway changes frequently coexist, theoretically allowing dysregulation of both the MAPK and PI3K pathways at the same time (8). As a result, PI3K inhibitors may provide some benefit to patients with PTEN and/or AKT-mutant melanomas (9). KRAS is a crucial member of the RAS gene family, and somatic KRAS mutations are found in about 30% of human cancers, especially in lung, pancreatic, colorectal, and bile duct cancers (10). KRAS mutations have different specificities depending on tumor type, with KRAS G12V mutations most commonly found in ovarian cancer, while the least common in cholangiocarcinoma (11). A subset of germ-cell tumors in non-epithelial ovarian malignancies can develop KRAS-activating mutations, as well as other genetic changes such as KIT and MAPK (12). It has been demonstrated that the incidence of KRAS mutations in intrahepatic cholangiocarcinoma and extrahepatic cholangiocarcinoma is 45%-54% and 10%-15%, respectively (13). Furthermore, the patient with intrahepatic cholangiocarcinoma has the worst prognosis when accompanied by TP53 and KRAS mutations (14). Shim et al (15)explored the genetic mutation characteristics of acrobatic malignant melanoma in the Korean population, and the results revealed that the mutation rates of KIT, NRAS, and BRAF genes were 8.5%, 4.3%, and 6.4%, respectively, indicating that acrobatic malignant melanoma shows more genomic changes and complicated chromosomal rearrangements. Moreover, an increase in KRAS expression was linked to melanoma progression, and wild-type KRAS was considered a potential target for melanoma (16). The KRAS mutation has been found to be present in a small percentage of primary bladder adenocarcinomas, is associated with the onset and prognosis of bladder cancer and can be used as a biomarker for bladder cancer surveillance and efficacy evaluation (17–19). Additionally, Donigian et al (20)confirmed that KRAS G12V mutation was present in bone malignant giant cell tumors. Although there are few studies on KRAS mutation-associated MPMNs, and the primary function of KRAS mutations is unclear, we hypothesize that KRAS mutations may be one of the causative factors of four primary tumors in this case, but the precise mechanism needs to be further studied.

In this case, bone tumors, bladder malignancies, and acral malignant melanoma or intrahepatic cholangiocarcinoma did not occur synchronously, while the histopathology of bone tumors is unclear, and it is also unclear whether adjuvant chemotherapy is given after surgery. However, adjuvant chemotherapy was carried out after surgery for bladder malignancy, but the specific medication is unknown. Given the patient’s denial of immune-related diseases, history of toxic exposure, and family history of malignancy, combined with the missense mutation in exon 2 of the KRAS gene, malignant melanoma or intrahepatic cholangiocarcinoma may be associated with adjuvant chemotherapy for bladder cancer and KRAS mutations (21). Moreover, cancer data from National Cancer Institute show that about 16% of all new cancer cases have a second primary malignancy (22), suggesting that the first primary malignancy and its treatment, such as radiation therapy, chemotherapy, and endocrine therapy, are important factors in the second primary malignancy or MPMNs.

Acral melanoma is a rare subtype of melanoma that typically develops on non-hairy skin, such as the palms, soles, and nail bed (23). For the present case, enlarged excision and toe amputation were performed after a definitive diagnosis to prevent local recurrence and improve prognosis (24). Immunotherapy has dramatically advanced in the treatment of malignant melanoma in recent years, significantly improving patient outcomes. Approximately 3% of melanomas have no identifiable primary, which is referred to as melanoma of unknown primary (MUP). Patients with MUP have been shown to have better outcomes than those with stage-matched melanoma of known primary (MKP), most likely due to their increased immunogenicity, as evidenced by immunologically mediated primary site regression. As a result, patients with melanoma of unknown primary site who receive immunotherapy are likely to have better outcomes than those with melanoma of known primary site (25). Ipilimumab, a fully humanized monoclonal antibody against CTLA-4, was the first immune checkpoint inhibitor approved for advanced malignant melanoma, receiving FDA approval in 2011 (26). Toripalimab was approved by the FDA in 2018 for solid tumors such as malignant melanoma (27), with an objective response rate (ORR) of 20.7% for advanced malignant melanoma (mostly Acral and mucosal melanoma) (28), while Pucotenlimab(HX008), an anti-PD-1 humanized IgG4 monoclonal antibody yielded an up to 20.2% of ORR for locally advanced or metastatic melanoma (29). Checkmate 238 study showed that nivolumab had a longer recurrence-free duration (RFS) and a lower incidence of high-grade adverse events than ipilimumab in resected stage III or IV melanoma (30). Another study discovered that pembrolizumab was both safe and effective in treating resected stage III melanoma. Nivolumab and pembrolizumab have been approved for adjuvant therapy of resected stage III melanoma in light of these trials (31–33). Nivolumab combined with Ipilimumab or nivolumab alone significantly improved RFS compared to placebo in patients with resected or unresected stage III-IV melanoma, while nivolumab combined with Ipilimumab or nivolumab alone significantly improved OS compared to Ipilimumab, and nivolumab combined with Ipilimumab had better efficacy (34, 35). At present, there is no consensus on the sequential selection of targeted therapy and immunotherapy in melanoma. The clinical studies are frequently based on tumor burden to select targeted and/or immunotherapy, and there is a lack of evidence for large-scale prospective sequential studies on targeted and immunotherapy. Numerous clinical studies, such as DREAMseq (36)and SECOMBIT (37), have been conducted in response to current clinical issues. According to the latest study (38), the median PFS and ORR of patients with advanced Acral melanoma treated with first-line Camrelizumab combined with Apatinib and temozolomide were 18.4 months and 66.7%, respectively, which is expected to become the standard treatment strategy for advanced acral melanoma.

Cholangiocarcinoma is a highly malignant tumor with a median survival time of fewer than two years (39). GP (gemcitabine/cisplatin) regimen is currently regarded as the standard first-line chemotherapy strategy for advanced cholangiocarcinoma, according to the findings of phase III clinical trial (ABC-02) (40). The multicenter Phase II clinical trial showed that the first-line treatment using Camrelizumab combined with FOLFOX4 (fluorouracil/leucovorin/oxaliplatin) or GEMOX (gemcitabine/oxaliplatin) for patients with cholangiocarcinoma resulted in a disease control rate (DCR) of 67.4%, providing new options for the treatment of advanced cholangiocarcinoma (41). Furthermore, the KEYNOTE-966 study also suggested that immunotherapy in combination with chemotherapy has potential value in exploring the comprehensive treatment of cholangiocarcinoma (42). There is currently no standard treatment regimen for multiple primary tumors. Given the coexistence of acral melanoma and intrahepatic cholangiocarcinoma in the present patient, as well as the clinical trial results of malignant melanoma or cholangiocarcinoma, the therapeutic regimen needs to take into the Coexisting tumors. So, a systemic treatment regimen combining GP with Camrelizumab, followed by Camrelizumab maintenance, was used, and it achieved satisfactory efficacy with a 1-year survival time.

The histological type of bone tumor and whether adjuvant therapy is administered postoperatively, as well as the histological type of bladder malignancy, are unknown, and these are the case report’s main limitations. As a result, four primary cancer diagnoses are debatable.

## Conclusion

In summary, the pathogenesis of MPMNs has not been elucidated, and its potential causes are involved in second primary malignancies induced by anti-tumor therapy such as radiation therapy, chemotherapy, and endocrine therapy, as well as gene mutations. NGS in clinical management is necessary to identify the associated gene mutations. The treatment of multiple primary tumors or MPMNs needs to take into account the coexisting tumors, and systemic treatment including immunotherapy and targeted therapy can improve the prognosis of patients with MPMNs. In the present case, the diagnosis and treatment strategy of four primary tumors is expected to provide an important foundation for the clinical diagnosis and treatment of such tumors.

## Data availability statement

The original contributions presented in the study are included in the article/**Supplementary Material**. Further inquiries can be directed to the corresponding author.

## Ethics statement

The studies involving human participants were reviewed and approved by the Ethical Committee of the Second Hospital Affiliated with Lanzhou University. Written informed consent was obtained for the publication of this case report.

## Author contributions

X-YM: writing and literature review. KT: clinical data collecting and literature review. P-FS: contributed to the writing and revision of the manuscript. Authors X-YM and KT both made an equal contribution to this work. All authors contributed to the article and approved the submitted version.
