# Supplementary material for: Multiple primary malignant neoplasm: Case report and comprehensive literature review
Source: Front Oncol. 2023 Jan 4;12:1090634. doi: 10.3389/fonc.2022.1090634 (PMC9846320; doi:10.3389/fonc.2022.1090634)
Supplement: Supplementary file 1 [file DataSheet_1.docx]

CARE Supplementary materials

**Patient Information** **5c:** Medical, family, and psycho-social history including relevant genetic information： The patient has been suffering from hypertension for more than 40 years without a history of diabetes, coronary heart disease, and tuberculosis, the most hypertension is 160/90mmHg, well controlled by oral antihypertensive drugs. Underwent bone tumor surgery in left-hand middle finger 45 years ago, cholecystectomy for gallbladder stones 39 years ago, and bladder malignant tumor resection followed by adjuvant chemotherapy 15 years ago. The exact pathological diagnosis and systemic treatment are unknown at that time. Since then, the patient has lived in a good state without the clinical manifestations of tumor recurrence and metastasis. Deny a family history of malignancy.

**Timeline 7：**Historical and current information from this episode of care organized as a timeline

1. Presented to the Plastic Surgery Department of our hospital complaining of " the black mass on the bigger toe of the right foot for more than 4 years " on early March 5, 2021.
2. Excision of skin lesions was performed on March 8, 2021, and postoperative pathology diagnosed malignant melanoma of the extremity.
3. Toe amputation of the right toe under lumbar anesthesia on March 15, 2021.
4. March 17, 2021 PET/CT scan revealed a mild hypodensity mass in the lateral segment of the left hepatic lobe with a maximum SUV value of 12.0, which was diagnosed as a malignant tumor. In addition, enlarged lymph nodes were visible in the left parotid gland, hilar hepatic and retroperitoneal regions, and FDG metabolism is elevated, which was considered as metastasis.
5. In mid-April 2021, she was admitted to our medical oncology department for chemotherapy 1 month after surgery for malignant melanoma. After admission, laboratory tests revealed CA199>1000.00U/ml and PET-CT revealed an occupying lesion in the liver, raising the probability of a gastrointestinal tumor. A liver aspiration biopsy was then conducted, and the pathology revealed intrahepatic cholangiocarcinoma. Serum NGS testing suggested a potentially clinically significant missense mutation in exon 2 of the KRAS gene, namely P.G12V.
6. From April 2021 to August 2021, 6 cycles of GP (gemcitabine hydrochloride 1.5 g, d1, 8 / cisplatin 40 mg, d1, 8) combined with anti-PD-1 inhibitors (Camrelizumab 200 mg, d1) system therapy.
7. Followed by 3 cycles of Camrelizumab (200 mg, q3w) maintenance therapy from September to October 2021, and efficacy evaluation (RECIST version 1.1) stabilized (SD) throughout treatment.
8. She was re-admission because of mild yellowing sclera in December 2021, laboratory tests showed abnormal liver function indicators such as DBIL 40.1 umol/L, IBIL 48.1 umol/L, ALT 205 U/L, and AST 181 U/L. MRCP indicated multiple liver metastases and biliary obstruction, and tumor progression was considered, so the 4th cycle of immune maintenance therapy was suspended. To improve liver function, transendoscopic retrograde cholangiography (ERCP) and biliary stenting were also carried out.
9. The tumor continued to progress 1 month after ERCP surgery, the patient and family refused to continue anti-tumor therapy.
10. Infectious shock and multiple organ failure caused the patient's death in March 2022.
